# Usefulness of cerebrospinal fluid presepsin (soluble CD14 subtype) as a new marker in the diagnosis of neurosurgical postoperative meningitis

**DOI:** 10.3389/fneur.2024.1429354

**Published:** 2024-07-18

**Authors:** Yutaka Fuchinoue, Kosuke Kondo, Yuki Sakaeyama, Chie Nakada, Sayaka Terazono, Syuhei Kubota, Masataka Mikai, Mituyoshi Abe, Shinji Ujiie, Toshisuke Morita, Nobuo Sugo

**Affiliations:** ^1^Department of Neurosurgery (Omori), Faculty of Medicine, Toho University, Tokyo, Japan; ^2^Department of Laboratory Medicine, Faculty of Medicine, Toho University, Tokyo, Japan

**Keywords:** presepsin, CSF presepsin, soluble CD14 subtype, meningitis, postoperative meningitis, neurosurgical postoperative meningitis

## Abstract

**Objective:**

To determine the usefulness of cerebrospinal fluid (CSF) presepsin in the diagnosis of neurosurgical postoperative meningitis (POM).

**Methods:**

The study included patients admitted to the Department of Neurosurgery, Toho University Medical Center Omori Hospital from May 1, 2020 to March 31, 2022 with suspected meningitis after neurosurgery who clinically required CSF sampling and patients who underwent CSF sampling for examination of idiopathic normal pressure hydrocephalus (iNPH). Participants were divided into a POM and a postoperative non meningitis (PONM) group based on the POM diagnostic criteria established for this study. The control group included patients from whom a CSF sample for iNPH was collected by tap test.

**Results:**

A total of 238 CSF samples were collected from 90 patients. There were 39 samples in the POM, 180 samples in the PONM, and 19 samples in the control group. CSF presepsin levels in the POM were significantly higher than in the PONM group (1764.5 and 440.9 pg./mL, respectively; *p* < 0.0001). The control group had CSF presepsin levels of 95.5 pg./mL. A cutoff value of 669 pg./mL for CSF presepsin in POM and PONM groups had 76.9% sensitivity and 78.3% specificity for the diagnosis of POM. In analyzes including only subarachnoid hemorrhage (SAH) cases (123 samples), CSF presepsin (1251.2 pg./mL) in the POM was significantly higher than in the PONM subgroup (453.9 pg./mL; *p* < 0.0001). The cutoff value for presepsin in CSF among patients with SAH (669 pg./mL) had 87.5% sensitivity and 76.6% specificity, similar to that of all patients.

**Conclusion:**

CSF presepsin is a useful marker in the diagnosis of neurosurgical POM even in patients with blood components, such as SAH. When POM is suspected, measurement of CSF presepsin may be recommended in addition to a general CSF examination.

## Introduction

The incidence of bacterial meningitis after neurosurgery is 0.3–25% (1–4). Particularly, patients with intraventricular and lumbar catheters are at risk of postoperative meningitis (POM) (5, 6) and nearly one third (10–27%) of patients with an external ventricular drainage have meningitis (7–11). In a previous study including 6,243 neurosurgical patients, patients with/without POM had a 3-month mortality rate of 13.7 and 4.7%, respectively (1). Moreover, a previous review of 334 craniotomies found a 29% mortality rate from POM (12). Thus, a prompt POM diagnosis is essential to improve patient outcomes (5).

POM symptoms include headache, nausea, malaise, altered consciousness, convulsions, neck stiffness, and other meningeal signs, as well as skin redness and tenderness associated with subcutaneous shunting and fever without other obvious foci of infection (13). However, it is difficult to diagnose POM based only on clinical symptoms; cerebrospinal fluid (CSF) analysis is a cornerstone in diagnosing meningitis. In fact, the diagnosis of bacterial meningitis caused by paranasal sinusitis or otitis media is routinely based on CSF cell counts, glucose, and protein concentration (14). However, the CSF cell counts and protein levels increase after neurosurgical operations for subarachnoid hemorrhage (SAH) or intraventricular hemorrhage due to bloody CSF (14). CSF cultures have a higher diagnostic performance but are limited by its low positivity rates, the presence of contaminating bacteria, and the long time required to obtain culture results (15).

Presepsin is a soluble CD14 protein composed of 64 amino acids (16). CD14 is a glycolipid-anchored membrane glycoprotein expressed on cells of the myelomonocyte lineage (17). CD14 binds to bacteria and is phagocytosed by granulocytes together with the bacteria. CD14 is then cleaved by intracellular digestive enzymes into presepsin, which is released into the blood (16). From an early stage of infection, presepsin is found at high concentrations in the blood of patients with sepsis, reflecting disease severity (18). Notably, presepsin is less susceptible to noninfectious inflammatory conditions such as trauma, burns, and surgery than other conventional sepsis markers such as procalcitonin and C-reactive protein (CRP) (19, 20). So far, few studies have used CSF presepsin for the diagnosis of POM in the field of neurosurgery (14, 21, 22); thus, we aimed to investigate the utility of CSF presepsin for diagnosing meningitis after neurosurgery.

## Methods

The study subjects were patients who were suspected post meningitis after neurosurgical operations at Toho University Medical Center Omori Hospital between May 1, 2020, and March 31, 2022, and clinically required CSF sampling. Cases for whom CSF was collected for the diagnosis of idiopathic normal pressure hydrocephalus (iNPH) were also eligible for inclusion in our final analysis. CSF was collected through a lumbar puncture or a lumbar or ventricular drain. Routine CSF tests, CSF culture, and CSF presepsin levels were evaluated in this study.

Based on the United States guidelines for bacterial meningitis and Ortiz’s report (6, 23), this study adopted the following diagnostic criteria for POM: A body temperature ≥ 38°C, serum CRP ≥ 6 mg/dL, CSF cell count ≥1,000/μL, CSF polymorphonuclear cell ratio ≥ 80%, CSF protein ≥100 mg/dL, CSF glucose ≤40 mg/dL, and a CSF glucose: serum ratio < 0.4. POM Diagnosis was based on meeting ≥4 of the above criteria, and comprehensive evaluation by ≥2 Japan neurosurgical society board certified neurosurgeons.

In this study, patients who developed postoperative meningitis were included in the postoperative meningitis case group (POM-C), while those who did not were the postoperative non meningitis case group (PONM-C). Patient CSF specimens satisfying postoperative meningitis diagnostic criteria were categorized as postoperative meningitis specimens (POM-S), and specimens not doing so as PONM-S. A patient with multiple CSF tests had each specimen categorized into either a POM-S or a PONM-S. We also evaluated general CSF data, blood examination data, and clinical findings on the same day as CSF presepsin levels were measured. CSF collected by the tap test by lumbar puncture for iNPH was used as a control group.

### CSF presepsin estimation

CSF samples were first centrifuged (3,000 rpm × 10 min at 4°C) and the supernatant placed into a container for measurement (measurement time: 19 min) with STACIA (manufactured by LSI Medience, Tokyo, Japan).

The measurement protocol was as follows: (1) 25 μL of sample diluent was added to a 25 μL of CSF and heated to 37°C for 3.5 min, (2) 50 μL of enzyme-labeled antibody reagent was then added to the mixture and heated to 37°C for 4.2 min, (3) subsequently, 50 μL of magnetic latex reagent was added to the mixture and heated to 37°C for 4.4 min, (4) the mixture was washed out and 100 μL of organic solution was added to the extract and warmed to 37°C for 2.7 min before measuring luminescence measured.

### Statistical analysis

The Kruskal–Wallis test and Steel–Dwass method were used to compare the three patient groups (POM-C, PONM-C, and iNPH). The chi-squared test or Mann–Whitney U test were used to compare categorical and continuous variables between sample groups (POM-S and PONM-S). Univariate analysis using logistic regression was performed using POM as dependent variable and age, sex, serum glucose, CSF presepsin, CSF mononuclear cell ratio and POM diagnostic criteria CRP, CSF cell count, CSF polymorphonuclear cell ratio, CSF protein, CSF glucose, and CSF glucose: serum ratio as independent variables. In addition, receiver operating curve (ROC) analysis was performed to calculate the cutoff values for POM-S and the PONM-S. A subgroup analysis was performed for patients with SAH to illustrate the diagnostic utility of Presepsin in the presence of blood in CSF. A linear mixed analysis was performed on postoperative changes in presepsin, CRP, and white blood cell counts (WBC) over time from postoperative day 1 to 9 for patients with SAH. Statistical significance was set at *p* < 0.05. IBM SPSS Statistics 26 (IBM Corp., Armonk, NY, United States) was used for data analysis. This study was approved by the Ethics Committee of Toho University Omori Medical Center (approval number: M19212).

## Results

### Participant selection

**Figure 1:**
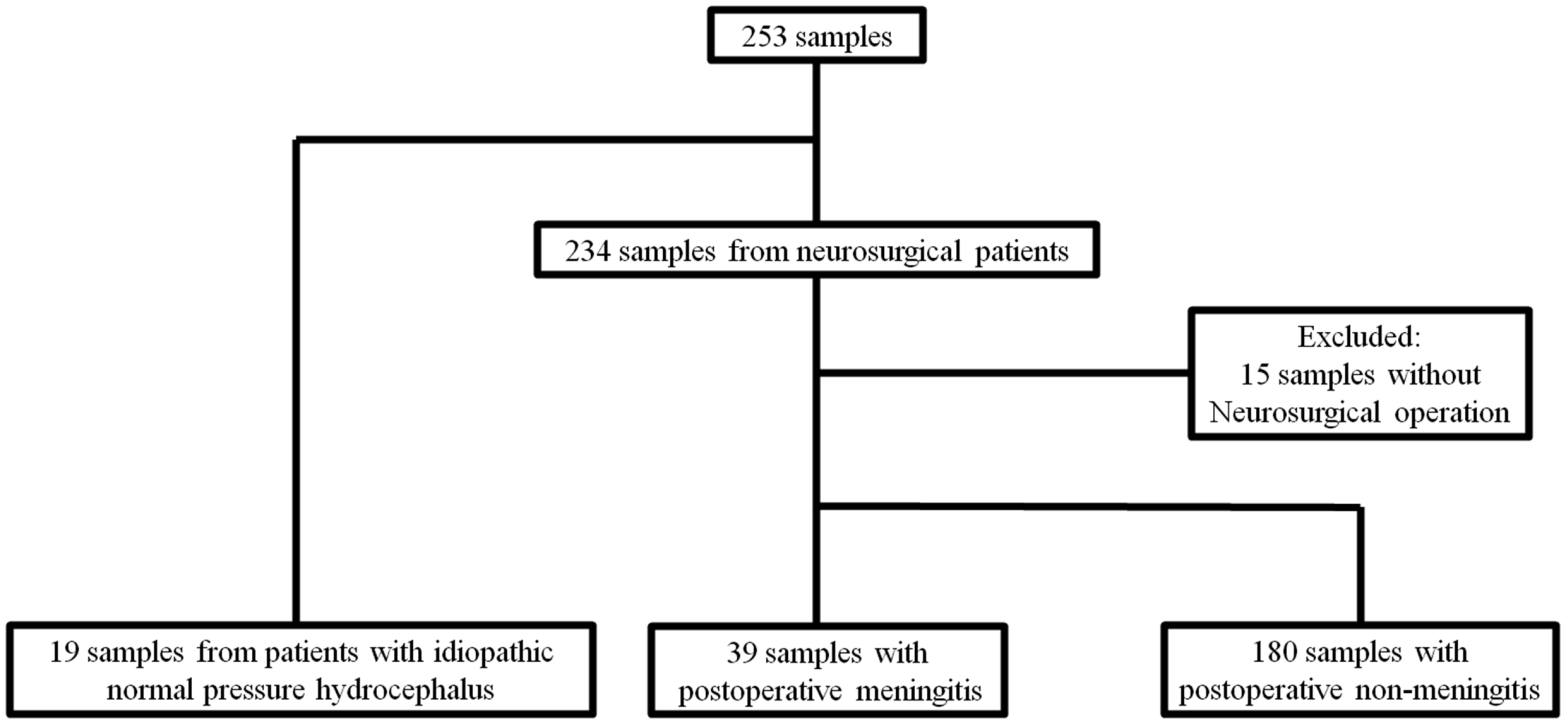
Flow diagram of the sample algorithm. A total of 253 samples from 90 patients including 234 samples from neurosurgical cases and 19 from the control group. In neurosurgical cases, after excluding 15 samples from patients that had not undergone operation, 39 and 180 samples were obtained in POM-S and PONM-S, respectively. iNPH, idiopathic normal pressure hydrocephalus; POM, Postoperative meningitis; POMN, Postoperative nonmeningitis.

A total of 253 samples from 90 patients including 234 samples from neurosurgical cases and 19 from the control group. In neurosurgical cases, after excluding 15 samples from patients that had not undergone operation, 39 and 180 samples were obtained in POM-S and PONM-S, respectively (Figure 1).

### Patient characteristics

The clinical characteristics of the POM-C, PONM-C, and iNPH control groups are shown in Table 1. The average age of the patients was 56.8 years in the POM, 60.4 years in the PONM, and 74.8 years in the control group. There was a significant difference (*p* < 0.01) between the three groups, but there no differences between the POM-C and PONM-C groups (*p* = 0.75). There was no significant difference in gender between the POM-C, PONM-C, and the control group (*p* = 0.68). The causative diseases in the POM-C were 12 cases of SAH, 6 cases of cerebral hemorrhage, 3 cases of brain tumor, and 1 case of others. In the PONM-C, there were 20 cases of SAH, 6 cases of cerebral hemorrhage, 15 cases of brain tumor, one case of hydrocephalus, seven other cases, and there was no significant difference in the causative disease between the POM-C and the PONM-C (*p* = 0.98). Treatment details for the POM-C, PONM-C included coiling, ventricular drainage, endoscopic surgery, clipping, shunt, tumor resection, others, and there was no significant difference between the 2 groups (*p* = 0.94) (Table 1). Next, the three groups were compared based on CSF samples. There were no differences among the sample groups according to disease procedure.

**Table 1:** Characteristics of patients who underwent neurosurgery at Toho University Medical Center Omori Hospital, Japan, 2020–2022 according to diagnostic criteria.

Patient characteristics	POM-C *N* = 22 *n* (%)	PONM-C *N* = 49 *n* (%)	Control *N* = 19 *n* (%)	*p-*value
Mean age (SD) in years	56.8 ± 17.6	60.4 ± 17.4	74.8 ± 6.5	<0.01
Sex				0.68
Male	13 (59)	26 (53)	13 (68)	
Female	9 (41)	23 (47)	6 (32)	
Diseases				0.98
Subarachnoid hemorrhage	12	20	0	
Intracranial hemorrhage	6	6	0	
Brain tumor	3	15	0	
NPH	0	1	19	
Others	1	7	0	
Surgical procedure				0.94
Coiling	9	10		
Ventricular drainage	8	5		
Endoscopic surgery	4	3		
Clipping	3	11		
Shunt	0	1		
Tumor resection	2	14		
Others	2	6		

POM-C, postoperative meningitis case group; PONM-C, postoperative nonmeningitis case group; Control group, idiopathic normal pressure hydrocephalus; NPH, normal pressure hydrocephalus.

### Sample characteristics

Patients’ sample characteristics are shown in Table 2. Patients’ body temperatures were significantly higher in the POM-S group than in the PONM-S group (*p* < 0.0001). CSF presepsin levels were significantly higher in the POM-S group than in the PONM-S group (1764.5 and 440.9 pg./mL, respectively; *p* < 0.0001). The CRP level (*p* < 0.0001), CSF cell count (*p* < 0.0001), CSF polymorphonuclear cell ratio (*p* < 0.0001), and CSF protein (*p* < 0.0001) level were significantly higher in the POM-S group than in the PONM-S. However, CSF mononuclear cell ratio (*p* < 0.0001), CSF glucose level (*p* < 0.0001), and CSF glucose: serum ratio (*p* < 0.0001) were significantly lower in the POM-S group than in the PONM-S. The mean values of CSF presepsin of patients with and without renal dysfunction (eGFR<60) were 475.0 and 703.8, respectively. These were not statistically significantly different (*p* = 0.58). There were 10 culture-positive cases. The isolates were *Enterococcus faecalis*, *Staphylococcus epidermidis*, *Staphylococcus capitis*, *Staphylococcus warneri*, *Staphylococcus aureus*, *Enterobacter aerogenes* were detected. The mean values of presepsin in culture-positive and culture-negative cases were 1,096 pg./mL (±793.0) and 603.6 pg./mL (± 1044.4), respectively, indicating that presepsin levels were significantly higher in culture-positive cases than in culture-negative cases (*p* = 0.02).

**Table 2:** CSF sample characteristics of patients who underwent neurosurgery at Toho University Medical Center Omori Hospital, Japan, 2020–2022.

Sample characteristics	POM-S *N* = 39 *n* (%)	PONM-S *N* = 180 *n* (%)	*p*-value
CSF collection			0.78
Spinal drainage	20 (51)	95 (52)	
Ventricular drainage	12 (31)	46 (26)	
Spinal tap	7 (18)	39 (22)	
Fever	38.6 ± 0.6	37.8 ± 0.9	<0.0001
Blood tests			
WBC (10^3^/mL)	11,159 ± 6496.3	9799.4 ± 3427.0	0.57
CRP (mg/dL)	9.4 ± 7.9	4.2 ± 5.5	<0.0001
Serum glucose (mg/dL)	139.4 ± 30.8	135.7 ± 43.8	0.27
CSF tests			
CSF presepsin (pg/mL)	1764.5 ± 1521.0	440.9 ± 484.1	<0.0001
CSF cell count (/μL)	3993.6 ± 7823.1	272.5 ± 524.9	<0.0001
% CSF mononuclear	0.3 ± 0.24	0.5 ± 0.29	<0.0001
% CSF polymorphonuclear	0.8 ± 0.22	0.4 ± 0.28	<0.0001
CSF protein (mg/dL)	334.7 ± 382.9	192.9 ± 353.2	<0.0001
CSF glucose (mg/dL)	42.0 ± 22.4	68.9 ± 19.9	<0.0001
CSF/blood glucose ratio	0.303 ± 0.157	0.525 ± 0.139	<0.0001

WBC, white blood cell count; CSF, cerebrospinal fluid; CRP, C-reactive protein; POM-S, postoperative meningitis sample group; PONM-S, postoperative nonmeningitis sample group.

### Factors predictive of POM

Univariate analysis with the presence or absence of POM as the objective variable showed significant differences in fever, CSF presepsin, CRP, CSF cell count, CSF mononuclear cell ratio, CSF polymorphonuclear cell ratio, CSF glucose, and CSF glucose: serum ratio (Table 3). The univariate analysis of this study included fever, CRP, CSF cell count, CSF polymorphonuclear cell ratio, CSF glucose, CSF protein, and CSF glucose: serum ratio, which were the diagnostic criteria for the POM. When multivariate analysis was performed without POM diagnostic criteria, CSF presepsin was the only predictive factor of POM (Table 4).

**Table 3:** Univariate logistic regression illustrating factors predictive of POM among CSF samples from patients who underwent neurosurgery at Toho University Medical Center Omori Hospital, Japan, 2020–2022 using diagnostic criteria as independent variables.

Variables	Partial regression coefficient	OR	95%CI	*p*-value
Age (≥65)	−0.0103	0.9898	0.9706–1.0094	0.31
Sex	−0.4922	0.6113	0.3011–1.2411	0.17
Fever	1.1766	3.2434	1.9706–5.3381	<0.0001
CSF presepsin	0.0018	1.0018	1.0011–1.0024	<0.0001
WBC	0.0001	1.0001	1.0000–1.0001	0.08
CRP	0.1036	1.1091	1.0556–1.1654	<0.0001
Serum glucose	0.002	1.002	0.9942–1.0099	0.61
CSF cell count	0.0016	1.0016	1.0010–1.0021	<0.0001
% CSF mononuclear	−3.2044	0.0406	0.0085–0.1927	<0.0001
% CSF polymornuclear	5.2497	190.5172	26.5644–1366.3713	<0.0001
CSF protein	0.0008	1.0008	1.0000–1.0016	0.05
CSF glucose	−0.0823	0.921	0.8958–0.9470	<0.0001
CSF/blood glucose	−12.2176	0	0.0000–0.0003	<0.0001

WBC, white blood cell count; CSF, cerebrospinal fluid; CRP, C-reactive protein; POM, postoperative meningitis.

**Table 4:** Multivariate logistic regression illustrating factors predictive of POM among CSF samples from patients who underwent neurosurgery at Toho University Medical Center Omori Hospital, Japan, 2020–2022 excluding diagnostic criteria independent variables.

Variables	Partial regression coefficient	OR	95%CI	*p*-value
Age (≥65)	−0.1150	0.8941	0.4048–1.9627	0.7752
Sex	−0.2295	0.7949	1.7462–0.5676	0.5676
CSF presepsin	2.2073	9.0909	4.1160–20.0785	<0.0001
WBC	−0.2081	0.3966	0.3733–0.5997	0.5997

WBC, white blood cell count; CSF, cerebrospinal fluid; POM, postoperative meningitis.

### ROC analysis

ROC analysis showed a sensitivity of 76.9% and specificity of 78.3% for a cutoff value of 669 pg./mL for CSF presepsin levels in the POM-S and PONM-S groups. In addition, the area under the curve (AUC) for CSF presepsin was 0.86 (95%CI 0.79–0.92). This was the second highest value compared to the AUC of other parameters (fever, WBC, CRP, CSF cell count, CSF mononuclear cell ratio, CSF polymorphonuclear cell ratio, CSF protein, CSF glucose, and CSF glucose; serum ratio) (Table 5).

**Table 5:** AUC value for each parameter.

Variables	AUC	95%CI	*p*-value	Sensitivity	Specificity
Fever	0.81	0.74–0.88	<0.0001	0.85	0.69
WBC	0.55	0.44–0.66	0.3524	0.59	0.47
CRP	0.72	0.63–0.82	<0.0001	0.62	0.78
CSF presepsin	0.86	0.79–0.92	<0.0001	0.77	0.78
%CSF mononuclear	0.73	0.64–0.82	<0.0001	0.68	0.75
%CSF polymorphonuclear	0.83	0.75–0.90	<0.0001	0.73	0.82
CSF protein	0.76	0.70–0.83	<0.0001	0.79	0.64
CSF glucose	0.83	0.74–0.92	<0.0001	0.79	0.75
CSF/blood glucose ratio	0.87	0.79–0.94	<0.0001	0.82	0.85

AUC, area under the curve; WBC, white blood cell count; CRP, C-reactive protein; CSF, cerebrospinal fluid.

### Subgroup analysis for patients with SAH

We compared the clinical characteristics of patients with SAH with ≥1 sample confirming POM to those who did not. There were no significant differences in age, sex, and surgical procedure between the two patient groups (Table 6).

**Table 6:** Characteristics of patients who underwent neurosurgery who had SAH at Toho University Medical Center Omori Hospital, Japan, 2020–2022 (*n* = 32).

Patient characteristics	POM-C-SAH	PONM-C-SAH	*p*-value
	*N* = 12	*N* = 20	
	*n* (%)	*n* (%)	
Mean age (SD) in years	62.1 ± 14.9	64.9 ± 14.5	0.533
Sex			0.52
Male	7 (58)	7(35)	
Female	5 (42)	13(65)	
CSF collection			0.83
Spinal drainage	50 (74)	53 (81)	
Ventricular drainage	12 (18)	3 (5)	
Spinal tap	6 (9)	9 (14)	
Surgical procedure			0.2
Coiling	9 (69)	8 (40)	
Clipping	3 (23)	11 (55)	
Others	1 (8)	1 (5)	

SAH, subarachnoid hemorrhage; CSF, cerebrospinal fluid; POM-C-SAH, postoperative meningitis case group SAH only; PONM-C-SAH, postoperative nonmeningitis case group SAH only.

CSF samples were also examined for patients with SAH. Among patients with SAH, there were a total of 123 samples, of which 16 met the criteria for POM-S and 107 did not. Body temperature was significantly higher in the POM-S than in the PONM-S (*p* < 0.001). CSF presepsin levels in the POM-S and PONM-S averaged 1251.2 and 453.9 pg./mL, respectively (*p* < 0.0001). CRP level (*p* = 0.0014), CSF cell count (*p* < 0.0001), CSF polynuclear cell ratio (*p* = 0.02), and CSF protein level (*p* = 0.0069) were significantly higher in the POM-S than in the PONM-S. Conversely, CSF glucose level (*p* = 0.0002) and CSF glucose: serum ratio (*p* < 0.0001) were significantly lower in the POM-S than in the PONM-S (Table 7).

**Table 7:** CSF sample characteristics of patients who underwent neurosurgery with SAH at Toho University Medical Center Omori Hospital, Japan, 2020–2022.

Sample characteristics	POM-S-SAH *N* = 16	PONM-S-SAH *N* = 107	*p*-value
Fever	38.5 ± 0.6	37.8 ± 0.7	<0.01
Blood tests			
WBC (10^3^/mL)	11418.8 ± 3635.8	9993.5 ± 2934.8	0.20
CRP (mg/dL)	8.0 ± 5.9	3.7 ± 3.8	<0.01
Serum glucose (mg/dL)	148.8 ± 37.7	138.1 ± 43.1	0.13
CSF tests			
CSF presepsin	1251.2 ± 700.4	454.0 ± 397.2	<0.01
CSF cell count (/μL)	1870.5 ± 1392.2	347.8 ± 602.3	<0.01
% CSF mononuclear	0.4 ± 0.3	0.5 ± 0.3	0.13
% CSF polymorphonuclear	0.6 ± 0.3	0.5 ± 0.3	<0.05
CSF protein	256.5 ± 242.3	233.0 ± 427.5	<0.01
CSF glucose (mg/dL)	46.2 ± 18.7	66.9 ± 19.2	<0.01
CSF/blood glucose ratio	0.3 ± 0.1	0.5 ± 0.1	<0.01

SAH, subarachnoid hemorrhage; WBC, white blood cell count; CSF, cerebrospinal fluid; CRP, C-reactive protein; POM-S-SAH, postoperative meningitis sample group SAH only; PONM-S-SAH, postoperative nonmeningitis sample group SAH only.

ROC analysis indicated a similar CSF presepsin cutoff value of 669 pg./mL for POM-S-SAH and PONM-S-SAH groups to that of all samples (sensitivity: 87.5%, specificity: 76.6%, area under the curve: 0.86, 95%CI 0.72–0.97).

**Figure 2:**
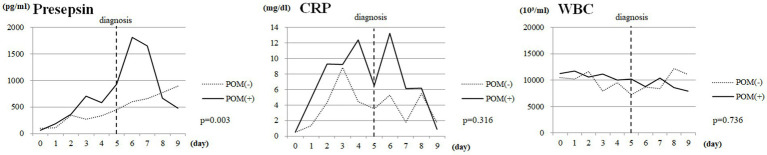
Changes over time in CSF presepsin, CRP, and WBC levels in SAH cases. Vertical dotted lines indicate the date of diagnosis of POM. CSF, cerebrospinal fluid; CRP, C-reactive protein; WBC, white blood cell count; SAH, Subarachnoid hemorrhage; POM, Postoperative meningitis; POMN, Postoperative nonmeningitis.

Finally, we evaluated changes in CSF presepsin, CRP, and WBC levels over time among SAH cases. The median diagnosis of postoperative meningitis was 5 days postoperatively. There were significant differences in CSF presepsin levels over time between the POM-S and the PONM-S (*p* = 0.03); but no differences between groups in CRP levels and WBC (Figure 2).

## Discussion

To the best of our knowledge, only four articles measured CSF presepsin levels for meningitis, one involving predictions (14, 21, 22, 24). Among those reports, the presepsin level in normal CSF was 50–100 pg./mL, increasing to 640.8 pg./mL in cases with bacterial meningitis demonstrating the usefulness of CSF presepsin level measurement for the diagnosis of bacterial meningitis (21). Further, in the that report, the CSF presepsin cutoff of 321 pg./mL did not vary with the presence or absence of blood in the CSF (21). However, these reports focused on community-acquired bacterial meningitis. The only report that measured CSF presepsin of POM is that of Zheng et al. They reported a CSF presepsin cutoff value of 1257.4–1276.2 pg./mL for POM and ventriculitis in neurosurgery, although they excluded patients with SAH (22). SAH is considered a disease with a high risk of POM because ventricular, cisternal, and spinal drains are often inserted to prevent spasm and to control intracranial pressure. Blood contamination in the CSF makes the diagnosis of bacterial meningitis difficult because it increases cell numbers in the CSF, and blood cells consume glucose producing lactic acid (25). In addition, the diagnosis of meningitis is difficult in cases with bloody CSF, such as SAH. Our study is the first report to demonstrate the utility of CSF presepsin in the diagnosis of neurosurgical POM in all patients, including those with SAH.

CD14 is found on the plasma membrane of granulocytes, macrophages, and monocytes and plays an important role in regulating the immune response to endotoxins (26). The CD14 on the cell membrane is internalized by phagocytic cells together with bacteria, digested, and cleaved by intracellular digestive enzymes, and converted into presepsin. Thereafter, it is released extracellularly and measured (27). Presepsin has been used as a novel marker to diagnose sepsis since its discovery in 2004 (3). In 2008, “PATHFAST Presepsin” (LSI Medience Corporation, Tokyo, Japan) was developed for fully automated measurement of presepsin with a chemiluminescent enzyme immunoassay, and numerous clinical evaluations have been conducted (28, 29). A multicenter clinical trial was conducted by Endo et al. (30) in 2012, which showed comparable or better results against the existing markers procalcitonin, IL-6, and CRP in the diagnosis of sepsis. The presepsin levels quantified during Gram-positive bacterial infection do not vary from those in Gram-negative bacterial infections (14, 30). Other common sepsis markers are CRP, procalcitonin, and interleukin-6 (IL-6). Procalcitonin is produced by cells stimulated by IL-6, but as protein synthesis is required for IL-6 production, this delays procalcitonin detection in the blood. Conversely, presepsin blood levels increase 2 h after infection peak in 3 h (19, 20, 31).

In this study, comparing the AUC of CSF presepsin to other parameters, CSF presepsin had the second highest value. This suggests that CSF presepsin is superior to routine parameters in detecting POM. It also had the third highest specificity. This suggests that the addition of CSF presepsin to existing parameters may provide a more accurate diagnosis of POM. In addition, markers other than CSF presepsin may be difficult to prove that the patient has POM on their own, while CSF presepsin, which is not affected by inflammation or bleeding associated with surgical invasion, may be able to diagnose POM on its own. During the postoperative course of cases with SAH, CSF presepsin levels peaked on the seventh postoperative day and were significantly higher in cases with POM than in cases with PONM. By contrast, CRP values showed a bimodal peak on postoperative days 3–4 and 6–7 but did not differ between POM and PONM cases. WBC counts did not change over time in both groups. In Figure 2, presepsin showed an upward trend from the date of diagnosis, whereas CRP reached its first peak before the date of diagnosis of POM. This suggests that CRP is rising due to non-infectious influences such as surgical invasion. In contrast, presepsin unimodal peak possibly reflects postoperative infection only as it is not affected by the surgical invasion. These results suggest that CSF presepsin may be a more accurate diagnostic marker for POM than other inflammatory markers. In our study, the CSF presepsin level in the control group (95.5 pg./mL) was comparable to the normal CSF presepsin (50–100 pg./mL) reported by Abudeev et al. (21). The cutoff of 669 pg./mL obtained in the present study lies within the range of the CSF presepsin levels described for POM in previous studies (625–1276.2 pg./mL) (14, 22).

### Limitations

Our study has some limitations. First, it was conducted at a single center, which limits its generalizability. Second, our sample size may limit group comparisons. However, a significant difference was obtained between the POM-S and the PONM-S groups. Nevertheless, future research should include more patients in a multicenter study. Third, blood presepsin and CSF lactate levels were not measured. However, CSF lactate levels are considered Grade D by the Infectious Diseases Society of America as they may be elevated by anaerobic metabolism, and metabolism by leukocytes in the CSF (23).

## Conclusion

CSF presepsin is a useful marker in the diagnosis of neurosurgical POM and in cases of SAH or when there is a blood component in the CSF. When POM is suspected, measurement of CSF presepsin may be recommended in addition to a general CSF examination.

## Data availability statement

The raw data supporting the conclusions of this article will be made available by the authors, without undue reservation.

## Ethics statement

The studies involving humans were approved by the Ethics Committee of Toho University Omori Medical Center. The studies were conducted in accordance with the local legislation and institutional requirements. The participants provided their written informed consent to participate in this study.

## Author contributions

YF: Conceptualization, Data curation, Formal analysis, Investigation, Methodology, Project administration, Validation, Writing – original draft, Writing – review & editing. KK: Data curation, Investigation, Methodology, Validation, Writing – review & editing. YS: Data curation, Investigation, Methodology, Validation, Writing – review & editing. CN: Data curation, Investigation, Methodology, Validation, Writing – review & editing. ST: Data curation, Investigation, Methodology, Validation, Writing – review & editing. SK: Data curation, Investigation, Methodology, Validation, Writing – review & editing. MM: Data curation, Investigation, Methodology, Validation, Writing – review & editing. MA: Data curation, Investigation, Methodology, Validation, Writing – review & editing. SU: Data curation, Investigation, Methodology, Validation, Writing – review & editing. TM: Writing – review & editing, Conceptualization, Data curation, Investigation, Methodology, Supervision, Validation. NS: Conceptualization, Data curation, Formal analysis, Investigation, Methodology, Project administration, Supervision, Validation, Writing – review & editing.
